# The Use of In Situ Simulation to Enhance COVID-19 Pandemic Preparedness in Obstetrics

**DOI:** 10.7759/cureus.12906

**Published:** 2021-01-25

**Authors:** Samantha Benlolo, Alysha Nensi, Douglas M Campbell, Caroline Assouad, Taryn S Taylor, Eliane M Shore

**Affiliations:** 1 Obstetrics and Gynaecology, St. Michael's Hospital, Unity Health Toronto, Toronto, CAN; 2 Paediatrics, St. Michael’s Hospital, Unity Health Toronto, Toronto, CAN; 3 Obstetrics and Gynaecology, London Health Sciences Centre, Schulich School of Medicine and Dentistry, London, CAN; 4 Obstetrics and Gynaecology, St. Michael Hospital, Unity Health Toronto, Toronto, CAN

**Keywords:** obstetrics, labor and delivery, pandemic preparedness, patient safety, simulation

## Abstract

Simulation’s benefits in medical education are well established. However, its use for pandemic preparedness in obstetrics is lacking. Management of obstetrical patients with suspected COVID-19 infection is a complex task with safety considerations for mother, fetus and healthcare workers. Implementation of new workflow algorithms to ensure safety is critical but is challenging to implement in real-time. We sought to improve pandemic preparedness by designing and deploying a high-fidelity simulation exercise involving the admission of a labouring obstetrical patient with suspected COVID-19 into a labour room, urgent transfer to the operating room and neonatal resuscitation. The creation of the simulation scenario was a multi-disciplinary effort with input from a focus group of key clinical stakeholders from within and outside of our centre to ensure clinical validity. Simulations were performed on the clinical unit during regular work hours so workflow could be observed in real-time with access to the equipment and personnel in which this clinical scenario would occur. We completed a total of 11 simulation sessions involving 42 participants. Feedback, obtained from debrief sessions and anonymous surveys, was categorized based on the human factors framework, and used as part of an iterative process to adapt, revise and improve the simulation scenario. The result of this iterative process was the creation of validated departmental infection control protocols that continue to be implemented through the second wave of the COVID-19 pandemic.

## Introduction

In situ simulation (ISS) is a tool used in healthcare settings to manage rare or complex clinical problems, and its benefits in medical education are well established [1]. Using simulation, trainees have the opportunity to bridge the gap between theory and clinical practice in a low-risk environment, without compromising patient safety [2]. More recently, simulation’s role has been expanded to evaluate and improve healthcare pathways and processes, creating a unique relationship between ISS and quality improvement [3,4].

Simulation is particularly relevant in novel, high-risk situations where the consequences of a flawed workflow algorithm can compromise patients’ and healthcare workers’ safety. The novel coronavirus (SARS-CoV-2) pandemic has presented us with such a situation. While most clinical practices have scaled down in response to the COVID-19 pandemic, the labor and delivery ward is unique as it requires uninterrupted coverage and cannot be suspended in order to modify patient care pathways. Without the opportunity to validate these changes as they are implemented, ISS is the critical piece in bridging the gap between policy and practice.

Management of obstetrical patients with known or suspected COVID-19 infection is a complex task with high-stakes considerations for the safety of the mother, fetus, and healthcare workers. Several studies have highlighted the significant impact COVID-19 infection has on maternal-fetal health. In a multi-national cohort study assessing the impact of COVID-19 infection in pregnancy, gestational age at the time of infection, need for ventilatory support in the mother and low birth weight was associated with adverse fetal outcomes [5,6]. In a national cohort of women in the United States, patients with COVID-19 infection in pregnancy had significantly higher rates of preterm delivery, preeclampsia, venous thromboembolism, myocardial infarction and death [7]. One of the most important principles to help reduce the spread of COVID-19 is infection control practices. While multiple society guidelines describe approaches to donning personal protective equipment (PPE) and workflows for emergent caesarean section (CS), implementation can be challenging in the clinical context [8]. In order to examine and analyze operational issues related to infection control practices on our obstetrical unit, we designed and implemented a high-fidelity ISS to replicate admission of a labouring obstetrical patient with known or suspected COVID-19 infection into a labour room, urgent transfer to the operating room (OR) for CS, and neonatal resuscitation.

The main objectives of the simulation included: (i) monitoring of team members applying principles of infection control, including use of PPE and awareness of departmental protocols, (ii) demonstration of situational awareness during the management of an obstetrical patient with suspected COVID-19 infection particularly with respect to respiratory compromise and (iii) utilization of non-technical skills such as shared mental models and closed-loop communication.

## Materials and methods

Participants included obstetricians, anesthetists, nurses, paediatricians, respiratory therapists (RT) and unit clerks. The simulations were performed over the course of eight weeks, on the clinical labour and delivery unit during regular day-time work hours so workflow could be observed in real-time with access to the equipment, personnel and physical space in which this clinical scenario would occur [9]. The simulation, orientation and pre-brief were led by one to two experienced simulation managers outside the clinical team. Each simulation was timed and observed by the simulation managers and included two embedded participants (laboring patient and partner) and an intubation torso with simulated vital signs (SimMon, Castle & Anderson 2018) to improve sensory fidelity.

The creation of the simulation scenario was a multi-disciplinary effort with input from a focus group of key clinical stakeholders from within and outside of our centre to ensure clinical validity. The debriefing strategy was based on a modified PEARLS framework [10] and participants were invited to participate in a voluntary, anonymous post-simulation survey. This was used to obtain feedback on its perceived utility, accuracy and effectiveness, and to suggest potential improvements.

## Results

We completed a total of 11 simulation sessions involving 42 participants (14 physicians, 28 nurses/RTs). Simulations lasted 20-60 mins and debriefing sessions lasted 20-40 minutes. Our initial simulation encompassed the entire patient flow pathway from arrival to transfer to the OR. The goal was not to meet all the objectives, but rather to test them against our patient flow algorithm in order to identify gaps in real-time. Following feedback from this initial session, we divided the initial simulation into three 'mini-sims' that each had a specific focus and set of objectives: (i) patient arrival to labor and delivery, (ii) patient transfer to the OR, and (iii) neonatal resuscitation. These 'mini-sims' were conducted in the same fashion as the full ISS but focused on one of the three above-mentioned goals. Due to the brevity of the mini-sim scenarios, we were able to carry them out two to three times each week. The entire patient flow pathway simulation (full ISS) was performed every two to three weeks. After completing four 'mini-sims' on patient arrival to labor and delivery, the first of our three 'mini-sim' scenarios, no further modifications to that aspect of our patient workflow algorithm were required. We then proceeded to focus on the second and third 'mini-sims', patient transfer to the OR and neonatal resuscitation, which required additional personnel (three nurses, two obstetrics providers, one anesthesia and one pediatrics provider) and physical space (labor room, OR and resuscitation room). The neonatal resuscitation mini-sim focused on neonatal transfer from the OR to the resuscitation room, specifically as it pertains to personal protective equipment requirements, communication between team-members during resuscitation and neonatal transfer to the neonatal intensive care unit with appropriate precautions (in an isolette with portable monitors and oxygen tank). It took four of these 'mini-sims' to create the final, most efficient and consistently reliable workflow. Over the eight-week time period, we ran the full simulation scenario three times. We tested and validated our most updated patient flow algorithm during the third full simulation, and presented this finalized algorithm at our department meeting as well as distributed the electronic version via email.

Following a critical analysis of the simulation sessions as well as the feedback provided, key issues were identified which encompassed safety, operational workflows and clinical logistics. Predominant themes included donning and doffing of PPE, the sequence of healthcare workers needed for patient transfer, equipment needed in the delivery room and OR, and communication systems for healthcare workers inside and outside of the delivery room and OR (Table 1).

**Table 1:** Observations and issues identified during simulation sessions and responses based on feedback and debrief sessions. Issues categorized based on the human factors framework [11].

Item	Observation	Response
Emergent factors - patient transfer to OR	The rushing of the donning process and uncertainty of donning order (due to acuity of the situation). The patient transferred to the OR area prematurely while team members still donning.	Patient flow algorithm adapted to include the most effective donning sequence (i.e., scrub nurse and baby nurse to be first donning pair). Large-print cognitive aids placed at each donning station to avoid relying on memory during an emergency scenario. Adapted patient flow algorithm ensures that the patient is last to arrive to the OR.
Physical environment - clustering at donning station	More than two donning buddies at donning station at one time resulting in crowding donning without a donning buddy leading to errors in PPE selection/application.	Creation of second donning station outside OR corridor to provide sufficient space for second donning pair. Separate donning and doffing training sessions made available for additional practice. Buddy system: buddies accountable for each other’s correct PPE application.
Human limitations - patient preparedness for the OR	Patient unprepared for OR on arrival leading to unnecessary delay - for example, failure to place foley catheter, IV line, EKG leads prior to patient transfer to OR.	Identification of steps to be taken in patient's room prior to patient transfer: insertion of foley catheter Insertion of peripheral IV placement of EKG monitors and BP cuff. Creation of to-do list placed in patient rooms to be used as a reference for team members under pressure.
Understanding people, goals and tasks - surgical safety checklist for emergency surgery during the pandemic	Failure to communicate the need for extra equipment leading to unnecessary door opening during surgery. Confusion regarding team members' roles and baby’s disposition.	Creation of adapted surgical safety checklist, including the anticipated need for additional equipment (e.g., Bakri balloon), clarification of team members roles, the designation of baby's disposition depending on clinical status (Appendix 1).
Physical environment - doffing process	Lack of hand sanitizer, linen bin and biohazard bin at the doffing station leading to improper doffing technique and the possibility of cross-contamination.	Touchless hand sanitizer installed at doffing station. Designated doffing linen and biohazard bins placed at the doffing station.

**Figure 1:**
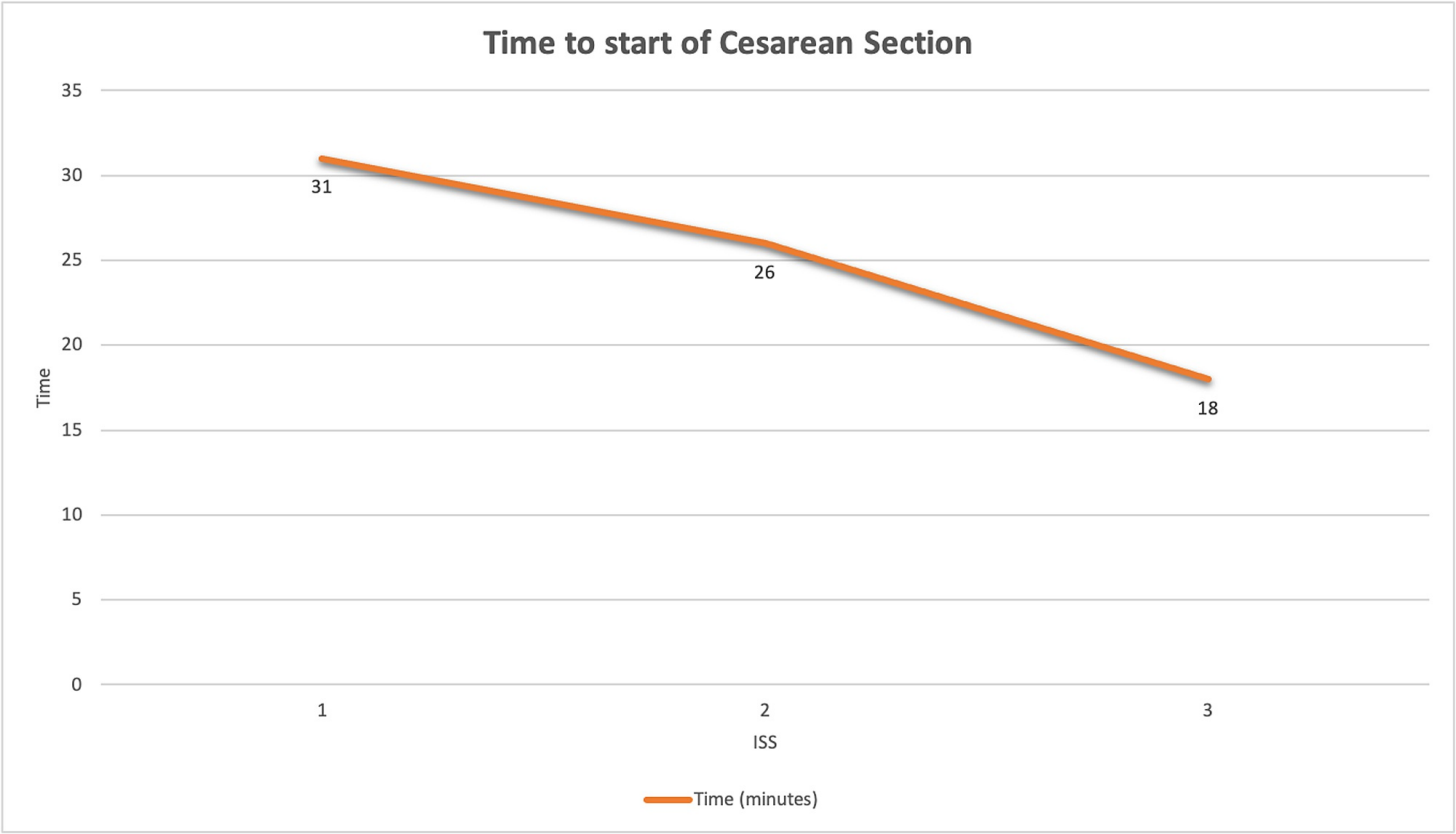
Time from the beginning of each of the three full ISS scenarios to the start of the simulated emergency cesarean section. ISS: in situ simulation.

Comments and feedback, categorized based on the human factors framework [11], were used as part of an iterative process to adapt, revise and improve both the simulation scenario and departmental infection control workflow protocols. Implementation of these strategies resulted in immediate improvements. By the final simulation, issues with infection control practices and sequence for OR transfer were resolved; specifically, the patient was not brought to the OR until the room and team were ready. Participants reported feeling more confident with donning and doffing, which made them less anxious about inadvertent exposures. Finally, the introduction of a pre-operative checklist to be performed in the patient’s labour room while waiting for the surgical team to don improved the expediency of starting the c-section, from 31 minutes to 18 minutes (Figure 1).

## Discussion

Our experience with ISS on the labor and delivery ward resulted in the creation of a complete, pandemic-specific, patient workflow protocol that places both patient and healthcare worker safety first. The simulation sessions were created in order to improve pandemic preparedness in the labor and delivery ward and had three objectives: (i) monitoring of team members applying principles of infection control, including use of PPE and awareness of departmental protocols, (ii) demonstration of situational awareness during the management of an obstetrical patient with suspected COVID-19 infection particularly with respect to respiratory compromise and (iii) utilization of non-technical skills such as shared mental models and closed-loop communication. These objectives were met following iterative adaptations of the simulation scenario and resulted in the creation of the final patient workflow protocol.

In addition to the latent safety threats that were identified and addressed, the more significant, foundational problem, was the presence of operational silos. Initially, various disciplines (obstetrics, nursing, infection control) were each creating their own operational workflows for patient care. The debrief sessions illustrated that the lack of communication and coordination between teams was threatening the development of an effective algorithm. Running the simulations allowed each of the teams to appreciate the roles, responsibilities and task lists of the others [12] and resulted in a meaningful integration of each specialty’s processes into the final workflow algorithm.

By testing our workflow protocols through the use of simulation, we were able to create a finalized and tested workflow algorithm ready for use for the entire department. By initiating this process early on in the pandemic, we were able to have a finalized protocol available prior to the surge in COVID-19 cases, significantly alleviating the anxiety of our healthcare workers. Additionally, we have been able to use the workflow for the second wave of the COVID-19 pandemic.

Due to the time constraints posed by the ongoing pandemic, we were limited in our ability to create a robust methodology a priori, evaluate the impact of our simulations with respect to adherence to PPE protocol, or compare changes in our process to a unit without ISS. Our aim for future iterations of in situ simulations on our unit is to evaluate the impact of simulation on pandemic preparedness and adherence to PPE protocol, by comparing clinical outcomes (time from decision to surgery start, video of PPE donning and doffing) between simulation participants and matched controls.

## Conclusions

The nature of obstetrics lies in its unpredictable potential for acuity and is what draws many obstetricians to the field. Simulation, in contrast, provides the experience of an acute situation in a safe environment, without risk or consequence. It has been integral to obstetrical emergency preparedness under usual circumstances, and considerably more so in the context of a global pandemic. In our experience, ISS allowed for uninterrupted workflow on the labour and delivery ward while simultaneously serving to inform, test and adapt clinical pathways to ensure patient and healthcare worker safety. This approach resulted in effective patient flow algorithms, improved communication and strengthened collaboration between multidisciplinary team members.

## Appendices

Surgical safety checklist - COVID-19

Brief

Surgeon:

❏   Patient Name and DOB or MRN:

❏    COVID-19 category: ❏ Negative ❏Suspected ❏ Positive

❏    Surgical Procedure:

❏    Pertinent Medical History:

❏    Allergies:

❏    Preoperative medications:

❏    Antibiotics:

❏    Thromboprophylaxis (medical or mechanical):

❏    GERD prophylaxis:

❏    Clarify roles as applicable - scrub nurse, circulating, baby nurse, paediatrics, anesthesia, RT, runner, baby monitor if available.

❏    Ensure all members are wearing the correct PPE level:

❏    Delayed cord clamping: Y or N

❏    Placenta to the path: Y or N

❏    Any additional specimens (ex: fallopian tubes):

❏    PPH risk? If so, B Lynch suture and Bakri in OR:

❏    Additional equipment needs (e.g., tubal ligation, iodine prep): 

Anesthesia:

❏    Planned anesthesia and intubation risk:

❏    Ensure equipment options available:

❏    ASA status:

Nursing:

❏    Equipment confirmation:

❏    The planned location for patient recovery after the case:

❏    Designated nurse to help with patient transfer:

Pediatrics:

❏    Fetal concerns:

❏    Where will the baby be going and who will the baby be handed off to:
